# Exploring the correlation between corrective glucose treatment and long-term patient outcomes: a SHINE secondary analysis

**DOI:** 10.3389/fneur.2025.1567766

**Published:** 2025-05-15

**Authors:** Paul Horton, Vishal Patel, C. L. Hall, Karen C. Johnston, Yajun Mei, Ofer Sadan

**Affiliations:** ^1^H. Milton Stewart School of Industrial and Systems Engineering, Georgia Institute of Technology, Atlanta, GA, United States; ^2^Division of Neurocritical Care, Department of Neurology and Neurosurgery, School of Medicine, Emory University, Atlanta, GA, United States; ^3^Department of Neurology, University of Virginia, Charlottesville, VA, United States

**Keywords:** acute ischemic stroke, glucose variability, glycemic control, relative hypoglycemia, patient outcomes

## Abstract

**Introduction:**

Glucose control is an important aspect of acute ischemic stroke management. Although absolute glucose concentration remains the focus in clinical stroke care, glucose variability is increasingly recognized as a viable treatment target. To assess the relationship between acute post-stroke glycemic control parameters and patient outcomes, we reanalyzed the data from the first 8 h of treatment for patients in the Stroke Hyperglycemia Insulin Network Effort (SHINE) clinical trial, when glycemic variability is highest.

**Methods:**

In this secondary analysis of the SHINE dataset, the rate of glucose change during the first 8 h was evaluated for its association with patient outcomes, dichotomized as modified Rankin scale (mRS) 0–2 versus 3–6, using logistic regression and a linear mixed-effects model.

**Results:**

Unadjusted analysis of the glucose correction period during the first 8 h suggested that patients with mRS 3–6 had a faster glucose correction compared to those with mRS 0–2 (−8.9 and −6.7 mg/dL/h, *p* < 0.001). This finding remained statistically significant in both the intensive intervention group and the poorly controlled diabetic sub-group (glycosylated hemoglobin [HbA1c] ≥ 6.4). Mixed-effects models also indicated a significant difference in the rate of glucose change (1.9 mg/dL/h, *p* < 0.001) between outcome groups (mRS 0–2 versus 3–6) across both treatment and HbA1c sub-groups.

**Conclusion:**

Analysis of the first 8 h of the SHINE data suggests that early, rapid correction of glucose is associated with poor outcomes, particularly in the sub-group of patients with HbA1c ≥ 6.4. Further research is warranted to assess early glycemic correction as a possible personalized glucose management goal.

## Introduction

Glycemic dysregulation is a known predictor of patient outcomes following acute ischemic stroke (1, 2). Multiple studies demonstrated that glycemic-related parameters and conditions are correlated with both the risk of stroke itself and subsequent complications (3–5). However, whether glycemic control during the acute phase of stroke is a modifiable risk factor remains unclear. The Stroke Hyperglycemia Insulin Network Effort (SHINE) randomized controlled clinical trial investigated this question by assigning patients with acute ischemic stroke to one of two treatment regimens: standard versus intensive glycemic control using a pre-specified glucose correction protocol (6). The study intervention was initiated within 12 h of admission and continued for 72 h from randomization. The SHINE trial was stopped early for futility, with 1,151 participants enrolled. Both the primary and secondary analyses did not demonstrate the benefit of the intensive treatment at 90 days (7). *Post-hoc* analysis of this trial data did not show a benefit in the subgroup treated with endovascular treatment for stroke (8), but it did suggest differences among other subgroups. For example, black patients had overall worse outcomes compared to white patients. Although this correlation was strongest in the subgroup of black patients with normal glycosylated hemoglobin (HbA1C < 6.4), the overall mean HbA1c in this group was higher, suggesting higher mean and variability of glucose at baseline (9). However, a separate *post-hoc* analysis could not identify a correlation between HbA1c across the entire cohort or glucose variability (defined as standard deviation) and patient outcomes in the SHINE trial data (10).

Glycemic dysregulation is a sign of illness and a poor prognostic factor in a variety of clinical states. This has been demonstrated in chronic conditions, such as poorly controlled diabetes mellitus, as well as in acute illnesses, such as sepsis, stroke, and other severe injuries (11–14). Glucose variability—as a measure of dysregulation—has previously been shown to correlate with patient outcomes in a variety of critically ill patients (13), including in acute ischemic stroke (15–17). Correcting elevated glucose levels inherently leads to glucose variability. Rather than absolute glucose concentration, assessing the rate of correction could influence both the absolute glucose, the variability and deliver a more individualized treatment target in the setting of acute stroke. Furthermore, the impact of early initial glucose correction on outcomes in acute stroke is not yet established. In the SHINE trial, the protocol mandated correction of initial hyperglycemia into one of the two treatment targets. This correction created an approximate 8 h window during which elevated initial glucose values were brought into the respective control range (7). Studying the initial correction regimen could inform future trial designs.

We performed a secondary analysis of the first 8 h (initial glucose correction phase) of the SHINE dataset to understand the association between the rate of hyperglycemia correction and patient outcomes. The study hypothesized that faster glucose correction, leading to higher variability and relative hypoglycemia, would correlate with poor patient outcomes.

## Methods

This is a secondary analysis of the SHINE prospective, multicenter, randomized, clinical trial. Anonymized data were obtained from the National Institute of Neurological Disorders and Stroke (NINDS) archived clinical research database. The Emory University Institutional Review Board (IRB) has exempted the study from IRB approval or consent due to the anonymization of the dataset. The SHINE study protocol (6) and results (7) were previously published. Briefly, it included 1,151 hyperglycemic participants diagnosed with an acute ischemic stroke who were randomized into a standard (target blood glucose range 80–179 mg/dL) or intensive treatment group (target glucose 80–130 mg/dL). In the intervention group, glucose was measured on average every 1–2 h with real-time adjustments via an insulin drip, compared to every 3 h and intervention every 6 h with subcutaneous insulin for the control group, with escalation to bolus dosing at 48 h. The inclusion criteria consisted of a glucose threshold of >110 mg/dL for those with a history of type 2 diabetes and ≥150 mg/dL for those without. Additionally, the baseline National Institute of Health Stroke Severity score (NIHSS) was restricted to values between 3 and 22. Patients with known type 1 diabetes mellitus were excluded. The study intervention lasted for up to 72 h after randomization. Using a sliding dichotomized 90-day modified Rankin Score (mRS) as the primary outcome, the study did not demonstrate a difference between the standard and intensive treatment groups. All available glucose data were used for this analysis.

For the current analysis, the patient cohort was divided by the treatment group (intensive versus standard) and by the baseline glycosylated hemoglobin (HbA1c, <6.4% versus ≥6.4%), creating four subgroups (standard HbA1C < 6.4, standard HbA1C ≥ 6.4, intensive HbA1C < 6.4, and intensive HbA1C ≥ 6.4,). Additionally, we converted the 90-day mRS to a binary scale comparing 0–2 versus 3–6 outcome groups. Initially, the original SHINE trial defined a favorable outcome as a sliding dichotomy based on the baseline NIHSS. A favorable outcome is defined under three separate presentations: a baseline NIHSS of 3 to 7 with a 90-day mRS of 0, a baseline NIHSS of 8 to 14 with a 90-day mRS of 0 to 1, and a baseline NIHSS of 15 to 22 with a 90-day mRS of 0 to 2. We also included an additional analysis in which a 90-day mRS between 0 and 2 was considered favorable, and a score of 3 or above was considered unfavorable. Herein, the term “favorable” or “unfavorable” outcomes refer to the original SHINE sliding dichotomy definition. Otherwise, outcomes are grouped as mRS 0–2 or 3–6.

To generate a smoothed glucose response trend, we converted the data to the following format: $\left(t_{\mathrm{ij}},Y_{\mathrm{ij}}\right)$ ; where $Y_{\mathrm{ij}}$ is the j-th glucose measurement for participant i, taken at time $t_{\mathrm{ij}}$ , where time is relative to the start of the protocol with $t_{i1}=0$ .

The variables in our analysis include HbA1c, treatment group, initial glucose value, baseline NIHSS, rate of glucose change in the first 8 h, and other demographic factors listed in Table 1. The initial glucose value was the measurement that qualified the patient for randomization, prior to any treatment. For the logistic regression, we calculated the rate of change of the glucose response in the first 8 h using a least squares regression line. In this context, the rate of glucose change indicates how quickly a participant was brought into the control range for the trial. The first 8 h correspond to the population average time required to reach the control range from the elevated initial value.

**Table 1:** Demographic and clinical features of the patient cohort by subgroup.

Parameter	Entire cohort	Standard treatment		Intensive treatment	
		HbA1c < 6.4	HbA1c ≥ 6.4	HbA1c < 6.4	HbA1c ≥ 6.4
*n*	1,106	132	414	134	426
Sex (% female)	45%	54%	43%	52%	42%
Age (mean ± SD)	65.8 ± 13.1	68.9 ± 15.4	65 ± 12.5	69.2 ± 12.9	65.1 ± 12.5
Race					
White	64%	68%	63%	72%	60%
Black	29%	24%	28%	23%	33%
Asian	3%	3%	3%	2%	2%
Other	4%	5%	4%	2%	4%
Unknown	1%	0%	2%	1%	1%
Ethnicity: Latino (%)	15%	23%	15%	14%	15%
Hypertension	88%	85%	89%	81%	91%
Type 2 DM*	80%	47%	91%	42%	88%
Hyperlipidemia	59%	55%	60%	55%	63%
Atrial fibrillation	20%	24%	16%	28%	19%
Prior stroke	17%	14%	19%	15%	19%
Number of glucose observations per patient (mean ± SD)*	36.8 ± 18.0	24.7 ± 6.3	24.4 ± 6.1	46.7 ± 16.8	49.9 ± 17.4
NIHSS on admission (lower quartile, median, upper quartile)	7, 12, 5	8, 16, 5	7, 12, 5	8, 12, 5	7, 12, 4
Initial glucose value (mean ± SD)^a^	185.1 ± 68.3	138.4 ± 40.1	202.3 ± 69.2	140.0 ± 40.0	196.7 ± 68.3
Intravenous tissue plasminogen activator	63%	66%	62%	69%	64%
Mechanical thrombectomy	13%	17%	11%	14%	12%
Proportion of patients with favorable functional outcome (mRS ≤ 2)	48%	45%	47%	49%	47%
Time from onset to protocol (hours, mean ± SD)	8.99 ± 3.1	8.56 ± 2.8	9.04 ± 2.9	8.86 ± 3.4	9.12 ± 3.14

Overall, the groups are comparable, apart from the frequency of diabetes mellitus type 2 (DM2, related to stratification by HbA1c) and the number of glucose measurements (due to the difference in the study intervention). Data presented as mean ± SD for continuous variables, as percentage for proportions, and as median, upper quartile, and lower quartile for NIHSS. *p-value <0.05 by comparing all four subgroups.

a Initial glucose value was defined as the first measurement when the protocol started.

### Statistical analysis

Descriptive statistics were calculated as mean ± standard deviation for continuous parameters, as percentages for categorical ones, and as median, upper, and lower quartiles for the NIHSS. We used the Wilcoxon rank-sum test to compare the distribution of continuous dependent variables for the two outcome groups. We used a one-way ANOVA test for evaluating the means of the continuous variables (such as age, number of glucose measurements, and initial glucose), the Kruskal–Wallis test for the NIHSS, and a chi-squared test for a contingency table for the remaining variables. We created the smoothed glucose response trends using a locally weighted scatter-plot smoothing (LOWESS) model, which depicts the trends and relationships between variables (18, 19). We evaluated the rate of change and intercept of the glucose response trend using a linear mixed-effects model (20). For the binary outcome models, we evaluated the relationship between individual dependent variables and outcomes using univariate logistic regression. All statistical tests were evaluated using a 95% confidence interval (CI). Data cleaning and feature engineering were performed in Python (v3.9.13), while modeling, visualization, and statistical analyses were performed in R (v4.2.1).

## Results

### Patient cohort

The SHINE cohort contained a total of 1,151 patients, of which 1,106 patients were analyzed. Thirty-five subjects were excluded from the analysis due to a missing HbA1c, and an additional 10 were excluded due to not being assigned to a treatment group. Four hundred and twenty-six subjects were in the intensive treatment group and had a baseline HbA1c ≥ 6.4%. There were 414 subjects randomized to the control arm with an HbA1c ≥ 6.4%. An additional 134 and 132 subjects had a baseline HbA1c < 6.4% and were randomized to the intervention and control groups, respectively. A comparison between the groups is detailed in Table 1.

### Differences in the change in glucose stratified by the patient outcomes

**Figure 1:**
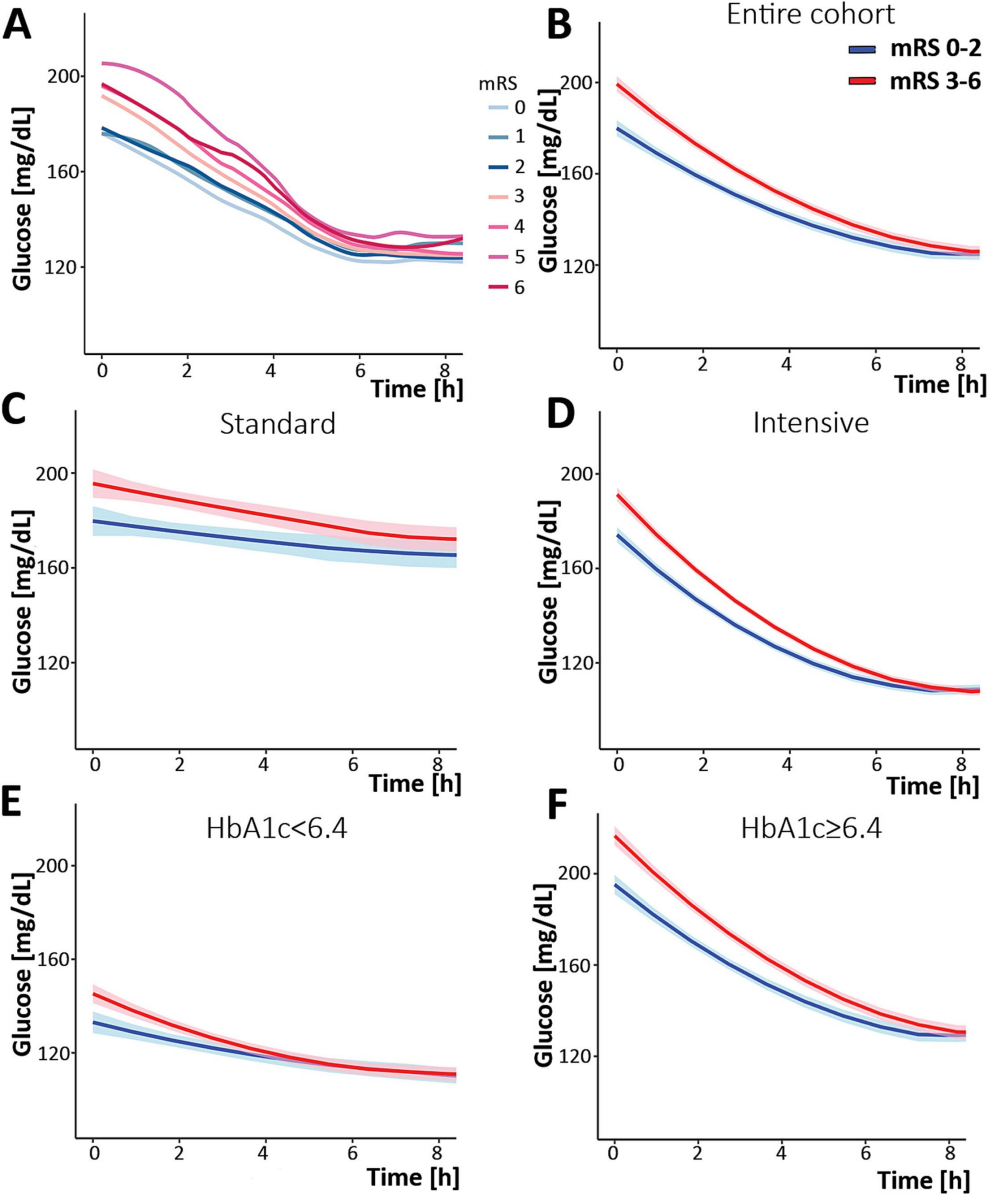
Change in serum glucose concentration over time during the first 8 h post-randomization. **(A)** analysis of the entire cohort separated by specific mRS, demonstrating a separation between mRS 0–2 and 3–6. **(B)** Analysis of the entire cohort separated by mRS: 0–2 (blue) and 3–6 (red). The same analysis was performed while separating the cohort into the group that was treated with the standard protocol **(C)**, the intervention (intense) protocol **(D)**, and according to HbA1c [**(E)** HbA1c < 6.4 and **(F)** HbA1c ≥ 6.4]. The shaded area describes the 95% confidence interval for the mean value. mRS, modified Rankin Scale.

According to the study’s original definition of favorable versus unfavorable outcomes, we noted a modest difference between the outcome groups for the rate of glucose change in the first 8 h (Supplementary Figure S1). Therefore, we analyzed the change in glucose according to individual mRS. Visual inspection of the changes demonstrated a separation when mRS is dichotomized to 0–2 and 3–6 (Figure 1A). Therefore, the rest of the analysis was performed using the conventional 0–2 and 3–6 dichotomy rather than the definitions used in the study. Upon linear fitting of the initial correction phase (first 8 h) by outcome, the rate of change coefficients were −6.7 and −8.9 mg/dL/h for the mRS 0–2 and 3–6 outcomes, respectively (*p* < 0.001, Figure 1B).

When the study population was further dissected by intervention, standard versus intensive, similar patterns were observed (Figures 1C,D). However, when dichotomizing patients by their HbA1c, a different pattern emerged. In the poorly controlled diabetic group (those with HbA1c ≥ 6.4), the pattern did not differ from the entire cohort: a separation between mRS 0–2 and 3–6 outcomes. This suggests glucose variability appears to impact outcomes in those with HbA1c ≥ 6.4. However, in the non-diabetic and well-controlled patients (HbA1c < 6.4), the two graphs overlapped (Figures 1E,F), suggesting that glucose variability was not associated with outcomes in these patients.

Upon combining the two parameters, namely the intervention arm and the HbA1c level, the group with elevated HbA1c, regardless of the treatment arm, exhibited a separation between trends by patient outcomes, while patients with normal HbA1c did not, regardless of the intervention.

### Examining the association between the initial 8-h glucose pattern and patient outcomes

**Figure 2:**
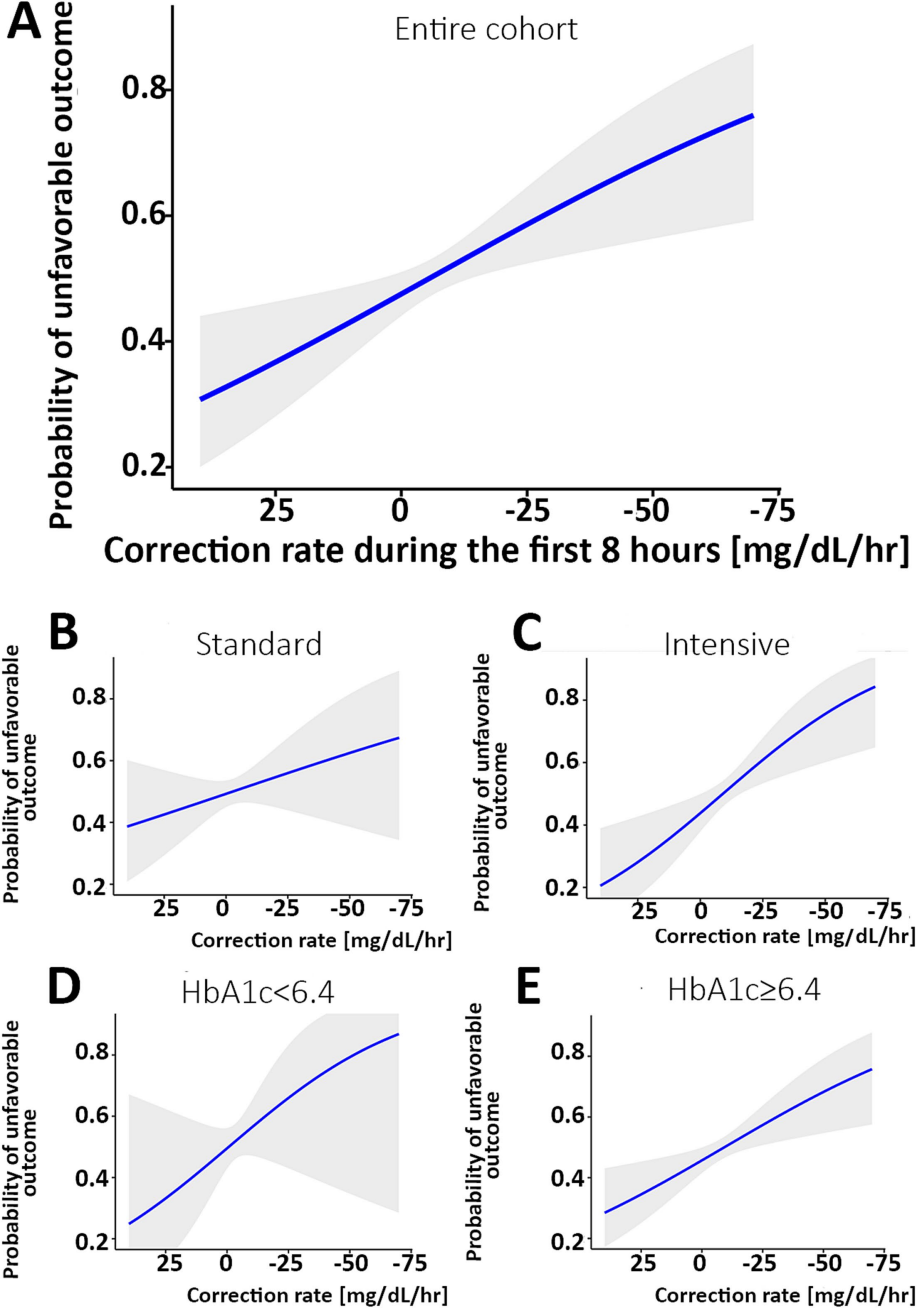
Correlation between the rate of glucose correction and the probability of an mRS 3–6 outcome. **(A)** The probability of an unfavorable outcome was higher as the rate of correction was faster in the entire cohort. When analyzing separately the group with the standard treatment **(B)** and intervention **(C)**, a steeper correlation is noted in the intervention group. When comparing patients with normal HbA1c [<6.4, **(D)**] versus elevated HbA1c **(E)**, the association is statistically significant with the higher HbA1c and not the normal HbA1c subgroup. The shaded area describes the 95% confidence interval for the mean value. The X-axis is reversed. mRS, modified Rankin Scale.

The relationship between the rate of change and the likelihood of worse outcomes is shown in Figure 2 and Table 2, where a faster decrease is associated with the mRS 3–6 (worse) outcome. The difference in the rate of glucose correction between the mRS 0–2 and 3–6 outcomes is notable when analyzing the entire cohort, suggesting a rapid initial correction of glucose is associated with the mRS 3–6 outcome (*p* = 0.009, Figure 2A). When analyzing the cohort by intervention, a faster glucose correction rate was associated with a higher risk of poor outcome, an effect limited to the intervention group (*p* = 0.001). This raises the potential for harm in patients who receive aggressive correction of their initial glucose (Figure 2C). The elevated HbA1c group demonstrated a similar pattern (*p* = 0.004, Figure 2E). There was a similar trend in both the standard treatment and the normal HbA1c groups, but these trends were not statistically significant (Figures 2B,D). However, this is likely due to an increased glucose threshold for the standard treatment group, which resulted in a smaller decrease from baseline regardless of outcome. The estimated effect size was larger for the normal HbA1c group compared to the elevated group, although it has a smaller sample size and, correspondingly, a larger confidence interval. A univariate logistic regression quantifying the relationship between the rate of change and poor outcome is detailed in Table 2.

**Table 2:** Univariate logistic regression using glucose slope to predict the stroke outcome of mRS 3–6 in the various sub-groups.

Subgroup	OR	95%CI	*p*-value
HbA1c ≥ 6.4	1.10	1.03–1.18	0.004
HbA1c < 6.4	1.26	1.00–1.62	0.061
Standard	1.07	0.96–1.18	0.219
Intensive	1.17	1.07–1.28	0.001

Note that the signs for the values of the glucose slope have been reversed in this table to align with the reversed x-axis in Figure 2, i.e., an OR>1 is predictive of an unfavorable outcome.

To avoid collinearity and isolate the association between the rate of correction and patient outcomes, we used a linear mixed-effects model (LME) to predict glucose values during the first 8 h. The model included input parameters such as the rate of glucose change and treated the intercept (initial glucose value) as a random effect between participants. The LME included outcome, rate of change, and interaction between the rate of change and outcome, corresponding to the effect the outcome has on the rate of change. The effects of race, treatment, and tissue plasminogen activator (tPA) on the rate were controlled through interaction terms. Table 3 shows the output of the model with coefficient values and corresponding *p*-values. When controlling for all the variables shown in Table 3, the results indicate that there is a 1.9 mg/dL/h faster rate of change for the participants in the worse outcome group (*p* < 0.001).

**Table 3:** Results of linear mixed-effects (LME) modeling on the glucose value within the first 8 h.

Predictor	Coefficient	*p*-value
Initial glucose	216.8	<0.01
Binary rankin	18.2	<0.01
Rate of change	−12.9	<0.01
Rate of change × Binary rankin	−1.9	<0.01
NIHSS	0.7	0.03
Rate of change × Treatment	9.9	<0.01
Rate of change × tPA	−0.8	0.03
Rate of change × White	1.4	<0.01
Age	−0.7	<0.01

×An interaction effect.

## Discussion

The SHINE trial was originally intended to evaluate the impact of intensive versus standard management of blood glucose on ischemic stroke patients. The SHINE trial did not demonstrate an improvement in patient outcomes based on the intensity of glucose management during the acute post-stroke phase. The focus of this paper was on glucose management in the first 8 h of the SHINE trial. We focused on the first 8 h for two reasons: first, this was the time of highest glucose variability, and second, we were interested in understanding how the rate of glucose correction could be a modifiable parameter in considering future clinical trial design. Indeed, an analysis of the time between 8 h and the end of the trial did not find a correlation between the binary mRS and glucose in this later period (data not shown).

This study found that a more rapid glucose correction rate was independently associated with worse functional outcomes. This finding was observed in the intensive management group, as might be expected given the *de facto* aggressive approach to glucose management in this arm. Interestingly, however, this finding was notable in the group with higher admission HbA1c, regardless of treatment intensity. These findings address the rate of glucose correction, and not the initial glucose level, which remains an important predictor of outcome, regardless of baseline HbA1c.

We further strengthened these findings with a linear mixed-effects model aimed at examining the correlation between multiple relevant parameters. This LME model further demonstrated an interaction between the rate of glucose correction, outcomes, and the predicted glucose measurements. Specifically, the results demonstrate that, even when addressing possible interactions or collinearity between key predictors, such as the assigned treatment group in the trial, thrombolytic treatment, race, the rate of change, and patient outcomes, the findings remained statistically significant and highly correlated with the actual glucose measurements. Overall, this analysis suggests that the rate of glucose correction could be a modifiable factor influencing blood glucose levels independently, with potential implications for patient outcomes.

This association between the rate of glucose correction during the first 8 h and worse outcomes was stronger in the group with poorly controlled diabetes than in the group with normal HbA1c. It remains unclear whether the striking association between the rate of glucose correction and outcomes in patients with elevated HbA1c is due to initially high glucose levels or because these patients’ baseline physiology allows higher glucose, such that a drastic correction would result in relative hypoglycemia. Relative hypoglycemia, defined as a decrease in blood glucose to levels lower than a patient’s average glucose based on their HbA1c, has recently been identified as a predictor of complications and poor outcomes in critically ill patients (21–23). According to the concept of relative hypoglycemia, the intervention arm of SHINE may have targeted a range too low for many of the patients. Baseline HbA1c informs relative hypoglycemia and stress hyperglycemia. Similarly, HbA1c can provide an estimate of the average blood glucose of a given patient. If the initial glucose is higher than the specific patient’s average, this could be related to stress hyperglycemia. Stress hyperglycemia was previously shown to be an independent predictor of poor outcomes following acute ischemic stroke in patients with or without diabetes (21, 24, 25). It remains unclear if the results herein stem from initial glucose and whether this is an inherent factor for the patient or a modifiable one. Initial elevated glucose in acute ischemic stroke is an established predictor for worse outcomes, as well as admission to the NIHSS, consistent with our results (24).

This study innovates by showing an association between the rate of glucose correction and patient outcomes, specifically in those with uncontrolled diabetes. The SHINE protocol (6), as well as previous similar studies, did not factor in the initial glucose levels or admission HbA1c when making protocol decisions. However, patients in the intervention arm received insulin drip-directed therapy with automated software to adjust for individual insulin sensitivity. This hypothesis-generating analysis suggests that perhaps a more personally tailored approach is needed when treating hyperglycemia post-stroke. Tight glucose management could be beneficial following stroke; however, the rate of correction may need to be slower in patients with higher baseline glucose or HbA1c levels and faster with those with stress hyperglycemia. This could be similar to the correction of a hypertensive emergency, which, per guidelines, should be performed in a stepwise manner according to the presenting blood pressure value (26).

This analysis has several limitations. First, this is a secondary analysis of an existing dataset, which was not designed to evaluate the rate of glucose change. Regardless, this dataset originated from a well-executed prospective randomized controlled study, which speaks to the high quality of data analyzed. Second, the SHINE study population primarily comprised patients with poorly controlled diabetes and cannot be generalized. Third, due to the difference between the treatment groups, the frequency of glucose measurements was different, which reduced the data density in the control group compared to the intervention one. This may have confounded the analysis of the rate of glucose change. Fourth, there could be important variables that were not measured herein. For example, although patients were randomized to certain treatment groups, not all patients achieved the desired glucose range, suggesting a functional crossover between the groups. Fifth, there is a risk of over-modeling due to the size of some of the subgroups analyzed (e.g., those with HbA1c < 6.4 within each treatment group). The models were developed using only this dataset and have not been internally or externally validated, raising the possibility that some relationships identified may be spurious. Sixth, as this study was hypothesis-generating, the multiple comparisons increase the risk of identifying relationships by chance. Finally, the original SHINE study used a sliding dichotomy to define a favorable outcome based on mRS at baseline, while this analysis used a simple dichotomy that was identified during the analysis. Considering the study results and limitations, future studies aimed at prospectively validating our results are necessary. Such studies may inform the optimal glucose management following stroke and may benefit from considering baseline patient physiology and the rate of glucose correction in their design. Medical care is slowly embarking on an era of more continuous glycemic measures with new transdermal technology that will provide more data on glucose response. Further studies with both outpatient and inpatient data will be needed to guide a better understanding and enable personalized treatments to improve patient outcomes.

## Conclusion

A reanalysis of the SHINE clinical trial data found that a high rate of initial hyperglycemia correction was associated with worse patient outcomes. This analysis was hypothesis-generating and highlights areas that warrant further investigation and validation. While initial glucose is a known predictor of outcome following stroke, these results suggest that a differential and personalized approach to correcting initial hyperglycemia may play a role in future post-stroke treatment strategies.

## Data Availability

Publicly available datasets were analyzed in this study. This data can be found at: https://www.ninds.nih.gov/current-research/research-funded-ninds/clinical-research/archived-clinical-research-datasets.
