# Supplementary material for: Exploring the correlation between corrective glucose treatment and long-term patient outcomes: a SHINE secondary analysis
Source: Front Neurol. 2025 May 15;16:1567766. doi: 10.3389/fneur.2025.1567766 (PMC12119312; doi:10.3389/fneur.2025.1567766)
Supplement: Supplementary file 1 [file Table_1.docx]

Supplementary Material


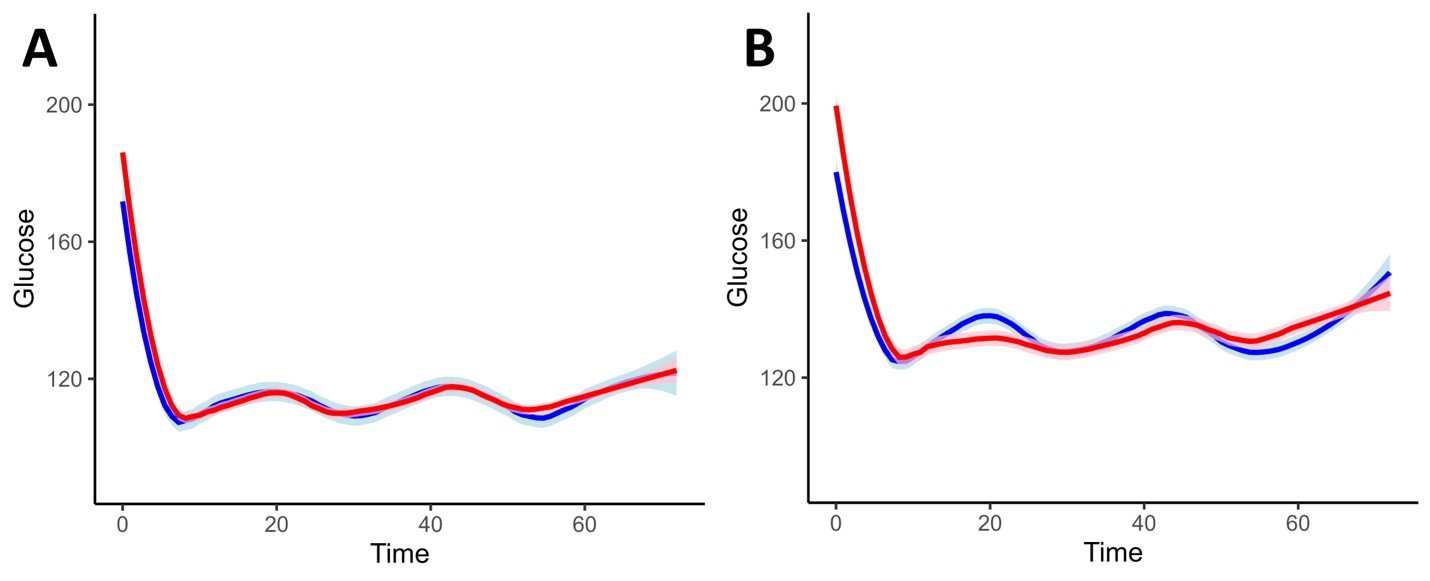


Supplementary figure S1: Change in glucose over the entire intervention timeframe (72 hours from randomization) by patient outcomes. (A) patient outcomes defined per the study protocol with a sliding dichotomy of modified Rankin Scale (mRS) based on the baseline mRS; A favorable outcome is defined under any of three conditions: a baseline NIHSS of 3 to 7 with a mRS of 0, a baseline NIHSS of 8 to 14 with a mRS of 0 to 1, and a baseline NIHSS of 15 to 22 with a mRS of 0 to 2. The analysis (Blue – favorable outcome, Red – unfavorable outcome) demonstrating a overlap between the groups. (B) Patient outcomes defined by a simple dichotomy (Blue – mRS 0-2, Red mRS 3-6) demonstrating separation between the groups especially in the first 8 hours of correction. The shaded area describes the 95% confidence interval for the mean value.
